# Supplementary material for: Are carbon emissions trading and green financial instruments synergistic? -Comprehensive quantitative research based on content analysis
Source: PLoS One. 2024 Mar 7;19(3):e0298601. doi: 10.1371/journal.pone.0298601 (PMC10919601; doi:10.1371/journal.pone.0298601)
Supplement: S1 Data — (ZIP) [file pone.0298601.s001.zip › Data/地级市Z_F工作报告.zip!/║■▒▒/╗╞╩»╩╨/└┤╫╘╣1⁄2╓┌║┼í╛┬φ┐╦╩2╛▌═°í┐_2012.docx]

各位代表：

　　 现在，我代表国务院，向大会报告政府工作，请各位代表审议，并请全国政协委员提出意见。

　　 一、2011年工作回顾

　　 过去的一年，面对复杂多变的国际政治经济环境和艰巨繁重的国内改革发展任务，全国各族人民在中国共产党领导下，同心同德，团结奋进，改革开放和社会主义现代化建设取得新的重大成就。国内生产总值47.2万亿元，比上年增长9.2；公共财政收入10.37万亿元，增长24.8；粮食产量57121万吨，再创历史新高；城镇新增就业1221万人，城镇居民人均可支配收入和农村居民人均纯收入实际增长8.4和11.4。我们巩固和扩大了应对国际金融危机冲击成果，实现了“十二五”时期良好开局。

　　 一年来，我们主要做了以下工作：

　　 （一）加强和改善宏观调控，遏制物价过快上涨，实现经济平稳较快发展。我们实施积极的财政政策和稳健的货币政策，坚持正确处理保持经济平稳较快发展、调整经济结构和管理通胀预期的关系，更加注重把握好政策实施的重点、力度和节奏，努力做到调控审慎灵活、适时适度，不断提高政策的针对性、灵活性和前瞻性。在全球通胀预期不断增强，国际市场大宗商品价格高位波动，国内要素成本明显上升，部分农产品供给偏紧的严峻形势下，我们把稳定物价总水平作为宏观调控的首要任务，坚持综合施策，合理运用货币政策工具，调节货币信贷增速，大力发展生产，保障供给，搞活流通，加强监管，居民消费价格指数、工业生产者出厂价格指数涨幅从8月份起逐月回落，扭转了一度过快上涨势头。下半年，世界经济不稳定性不确定性上升，国内经济运行出现一些新情况新问题，我们一方面坚持宏观调控的基本取向不变，保持宏观经济政策基本稳定，继续控制通货膨胀；一方面适时适度预调微调，加强信贷政策与产业政策的协调配合，加大结构性减税力度，重点支持实体经济特别是小型微型企业，重点支持民生工程特别是保障性安居工程，重点保证国家重大在建、续建项目的资金需要，有针对性地解决经济运行中的突出矛盾。我们坚定不移地加强房地产市场调控，确保调控政策落到实处、见到实效。投机、投资性需求得到明显抑制，多数城市房价环比下降，调控效果正在显现。我们高度重视防范和化解财政金融领域的潜在风险隐患，及时对地方政府性债务进行全面审计，摸清了多年形成的地方政府性债务的总规模、形成原因、偿还时限和区域分布。这些债务在经济社会发展中发挥了积极作用，形成了大量优质资产；也存在一些风险隐患，特别是部分偿债能力较弱地区存在局部性风险。我们认真开展债务清理整顿和规范工作，严格控制增量，积极稳妥解决债务偿还和在建项目后续融资问题。目前，我国政府性债务水平是可控的、安全的。总的看，我国国民经济继续朝着宏观调控预期方向发展，抗风险能力不断增强，呈现增长较快、价格趋稳、效益较好、民生改善的良好态势。

　　 （二）加快转变经济发展方式，提高发展的协调性和产业的竞争力。我们坚持有扶有控，促进结构调整和优化升级，增强发展后劲。

　　 巩固和加强农业基础。全面落实强农惠农富农政策，加大农业生产补贴力度，稳步提高粮食最低收购价，加强以农田水利为重点的农业农村基础设施建设，开展农村土地整治，加强农业科技服务和抗灾减灾，中央财政“三农”支出超过1万亿元，比上年增加1839亿元。农业全面丰收，粮食总产量实现了历史罕见的“八连增”，连续5年超万亿斤，标志着我国粮食综合生产能力稳定跃上新台阶。继续推进农村危房改造，解决了6398万农村人口的饮水安全和60万无电地区人口的用电问题，农村生产生活条件进一步改善。

　　 加快产业结构优化升级。大力培育战略性新兴产业，新能源、新材料、生物医药、高端装备制造、新能源汽车快速发展，三网融合、云计算、物联网试点示范工作步伐加快。企业兼并重组取得新进展。支持重点产业振兴和技术改造，中央预算投资安排150亿元，支持4000多个项目，带动总投资3000亿元。加快发展信息咨询、电子商务等现代服务业，新兴服务领域不断拓宽。交通运输产业快速发展，经济社会发展的基础进一步夯实。

　　 推进节能减排和生态环境保护。发布实施“十二五”节能减排综合性工作方案、控制温室气体排放工作方案和加强环境保护重点工作的意见。清洁能源发电装机达到2.9亿千瓦，比上年增加3356万千瓦。加强重点节能环保工程建设，新增城镇污水日处理能力1100万吨，5000多万千瓦新增燃煤发电机组全部安装脱硫设施。加大对高耗能、高排放和产能过剩行业的调控力度，淘汰落后的水泥产能1.5亿吨、炼铁产能3122万吨、焦炭产能1925万吨。实施天然林保护二期工程并提高补助标准，实行草原生态保护奖补政策，开展湖泊生态环境保护试点。植树造林9200多万亩。

　　 促进区域经济协调发展。深入实施区域发展总体战略和全国主体功能区规划。出台实施促进西藏、新疆等地区跨越式发展的一系列优惠政策。制定实施新10年农村扶贫开发纲要和兴边富民行动规划。区域发展协调性进一步增强，中西部和东北地区主要经济指标增速高于全国平均水平，东部地区产业转型升级步伐加快。城镇化率超过50，这是中国社会结构的一个历史性变化。胜利完成四川汶川特大地震灾后恢复重建任务，积极推进青海玉树、甘肃舟曲、云南盈江抗灾救灾和恢复重建工作。

　　 （三）大力发展社会事业，促进经济社会协调发展。各级政府加大对科技、教育、文化、卫生、体育事业的投入，全国财政支出2.82万亿元。

　　 持续提升科技创新能力。加强基础研究和前沿技术研究。实施国家科技重大专项，突破一些关键核心技术，填补了多项重大产品和装备的空白。天宫一号目标飞行器与神舟八号飞船先后成功发射并顺利交会对接，成为我国载人航天发展史上新的里程碑。

　　 扎实推进教育公平。深入贯彻落实教育改革和发展规划纲要。经过25年坚持不懈的努力，全面实现九年制义务教育。免除3000多万名农村寄宿制学生住宿费，其中1228万名中西部家庭经济困难学生享受生活补助。建立起完整的家庭经济困难学生资助体系。初步解决农民工随迁子女在城市接受义务教育的问题。推动实施“学前教育三年行动计划”，提高幼儿入园率。大力发展职业教育。加强中小学教师培训工作，扩大中小学教师职称制度改革试点，提高中小学教师队伍整体素质。首届免费师范生全部到中小学任教，90以上在中西部。

　　 大力加强文化建设。中央财政加大对文化惠民工程的支持，各地对公益性文化事业投入显著增加。扩大公共文化设施免费开放范围，服务面逐步拓展。文化体制改革继续推进，文化产业快速发展。文物保护、非物质文化遗产保护和传承取得重要进展。大力加强群众体育设施建设，全民健身活动蓬勃开展，体育事业取得新成绩。

　　 积极稳妥推进医药卫生事业改革发展。基本医疗保险覆盖范围继续扩大，13亿城乡居民参保，全民医保体系初步形成。政策范围内住院费用报销比例提高，重大疾病医疗保障病种范围进一步扩大。各级财政对城镇居民医保和新农合的补助标准由每人每年120元提高到200元。国家基本药物制度在政府办基层医疗卫生机构实现全覆盖，基本药物安全性提高、价格下降。公立医院改革试点有序进行。基层医疗卫生服务体系基本建成。基本公共卫生服务均等化取得新进展。

　　 （四）切实保障和改善民生，解决关系群众切身利益的问题。我们坚持民生优先，努力使发展成果惠及全体人民，促进社会公平正义。

　　 实施更加积极的就业政策。多渠道开发就业岗位，全力推动以创业带动就业，加强职业技能培训和公共就业服务体系建设。加大财政、税收、金融等方面支持力度，着力促进高校毕业生、农民工等重点人群就业。高校毕业生初次就业率77.8，同比提高1.2个百分点。农民工总量2.53亿人，比上年增长4.4，其中，外出农民工1.59亿人，增长3.4。

　　 积极调整收入分配关系。着力提高低收入群众收入。农村居民人均纯收入实际增速为1985年以来最高，连续两年快于城镇居民；各地普遍较大幅度调高最低工资标准；连续第7年提高企业退休人员基本养老金，全年人均增加1680元，5700多万人受益；进一步提高城乡低保补助水平以及部分优抚对象抚恤和生活补助标准，对全国城乡低保对象、农村五保供养对象等8600多万名困难群众发放一次性生活补贴；建立社会救助和保障标准与物价上涨挂钩的联动机制。扩大中等收入者所占比重。个人所得税起征点从2000元提高到3500元。降低900多万个体工商户税负。中央决定将农民人均纯收入2300元（2010年不变价）作为新的国家扶贫标准，比2009年提高92，把更多农村低收入人口纳入扶贫范围，这是社会的巨大进步。

　　 加强社会保障体系建设。社会保障覆盖范围继续扩大，全国参加城镇基本养老保险、失业保险、工伤保险和生育保险人数大幅增加。2147个县（市、区）实施城镇居民社会养老保险试点，1334万人参保，641万人领取养老金。2343个县（市、区）开展新型农村社会养老保险试点，3.58亿人参保，9880万人领取养老金，覆盖面扩大到60以上。解决了500多万名集体企业退休人员养老保障的历史遗留问题。将312万名企业“老工伤”人员和工亡职工供养亲属纳入工伤保险统筹管理。养老保险跨地区转移接续工作有序推进。社会保障体系不断健全，向制度全覆盖迈出重大步伐，这是推进基本公共服务均等化取得的重要成就。

　　 大力推进保障性安居工程建设。出台关于保障性安居工程建设和管理的指导意见，完善财政投入、土地供应、信贷支持、税费减免等政策，着力提高规划建设和工程质量水平，制定保障性住房分配、管理、退出等制度和办法。中央财政安排资金1713亿元，是2010年的2.2倍，全年城镇保障性住房基本建成432万套，新开工建设1043万套。

　　 努力维护社会公共安全。加强安全生产监管，做好重特大安全事故的处置、调查、问责工作。完善食品安全监管体制机制，集中打击、整治非法添加和违法生产加工行为。坚持以人为本、服务为先，加强和创新社会管理，着力排查化解各类社会矛盾，依法打击违法犯罪活动，保持社会和谐稳定。

　　 （五）深入推进改革开放，为经济社会发展注入新的活力和动力。我们按照“十二五”规划提出的改革任务，加大攻坚力度，推动重点领域和关键环节的改革。

　　 　完善公共财政体系特别是预算管理制度，把预算外资金全部纳入预算管理，扩大国有资本经营预算实施范围，深化部门预算改革，推进政府预算、决算公开，98个中央部门和北京、上海、广东、陕西等省市公开“三公经费”。在全国范围实施原油、天然气资源税从价计征改革，出台营业税改征增值税试点方案。把跨境贸易人民币结算范围扩大到全国，启动境外直接投资人民币结算试点，开展外商直接投资人民币结算业务。深化集体林权制度改革，启动国有林场改革试点，依法开展草原承包经营登记。推进水利建设管理体制改革，创新水资源管理体制。深化国有企业改革。启动实施电网主辅分离改革重组以及上网电价和非居民用电价格调整方案。基本完成乡镇机构改革。事业单位分类改革有序开展。

　　 我们坚持出口和进口并重，利用外资和对外投资并举，全面提升开放型经济水平。积极推进市场多元化战略，努力优化贸易结构。全年货物进出口总额3.64万亿美元，增长22.5，其中，出口增长20.3，进口增长24.9，贸易顺差进一步下降。实际使用外商直接投资1160亿美元，服务业和中西部地区比重提高。企业“走出去”步伐加快，非金融类对外直接投资601亿美元。积极参与国际和区域经济合作，多边双边经贸关系继续深化。

　　 我们在民主法制建设、国防和军队建设、港澳台工作和外交工作等方面，都取得了卓有成效的进展。

　　 过去一年的成绩来之不易，显示了中国特色社会主义的优越性和生命力，增强了中华民族的自豪感和凝聚力。这是以胡锦涛同志为总书记的党中央科学决策、正确领导的结果，是全党全军全国各族人民齐心协力、顽强拼搏的结果。我代表国务院，向全国各族人民，向各民主党派、各人民团体和各界人士，表示诚挚的感谢！向香港特别行政区同胞、澳门特别行政区同胞、台湾同胞和海外侨胞，表示诚挚的感谢！向关心和支持中国现代化建设的各国政府、国际组织和各国朋友，表示诚挚的感谢！

　　 我们也清醒地看到，我国经济社会发展仍然面临不少困难和挑战。从国际看，世界经济复苏进程艰难曲折，国际金融危机还在发展，一些国家主权债务危机短期内难以缓解。主要发达经济体失业率居高难下，增长动力不足，新兴经济体面临通货膨胀和经济增速回落的双重压力。主要货币汇率剧烈波动，大宗商品价格大幅震荡。国际贸易投资保护主义强化。从国内看，解决体制性结构性矛盾，缓解发展不平衡、不协调、不可持续的问题更为迫切、难度更大，经济运行中又出现不少新情况新问题。主要是：经济增长存在下行压力，物价水平仍处高位，房地产市场调控处于关键阶段，农业稳定发展、农民持续增收难度加大，就业总量压力与结构性矛盾并存，一些企业特别是小型微型企业经营困难增多，部分行业产能过剩凸显，能源消费总量增长过快。一些长期矛盾与短期问题相互交织，结构性因素和周期性因素相互作用，国内问题和国际问题相互关联，宏观调控面临更加复杂的局面。政府工作仍存在一些缺点和不足，节能减排、物价调控目标没有完成；征地拆迁、安全生产、食品药品安全、收入分配等方面问题还很突出，群众反映强烈；政府管理和服务水平有待提高，廉政建设亟需加强。

　　 我们一定要以对国家和人民高度负责的精神，采取更加有力的措施，切实解决存在的问题，努力把各项工作做得更好，决不辜负人民的重托。

　　 二、2012年工作总体部署

　　 今年是“十二五”时期承前启后的重要一年，也是本届政府任期的最后一年。我们要恪尽职守、锐意进取、攻坚克难，决不懈怠，交出一份人民满意的答卷。

　　 我国发展仍处于重要战略机遇期，在较长时期内继续保持经济平稳较快发展具备不少有利条件。工业化、城镇化和农业现代化快速推进，消费结构和产业结构升级蕴藏着巨大的需求潜力；经过30多年改革开放，我国发展建立了良好的物质基础和体制条件，宏观调控经验不断丰富，企业竞争力和抗风险能力明显提高；东部地区创新发展能力增强，中西部地区和东北等老工业基地发展潜力不断释放；经济发展的传统优势依然存在，劳动力资源丰富、素质提高；财政收支状况良好，金融体系运行稳健，社会资金比较充裕。世界经济政治格局正在发生深刻变化，和平、发展、合作仍然是时代潮流，总体上有利于我国和平发展。我们要坚定信心，善于运用有利条件和积极因素，继续抓住和用好重要战略机遇期，推动经济平稳较快发展，不断增强我国的综合国力和国际影响力。

　　 我们要高举中国特色社会主义伟大旗帜，以邓小平理论和“三个代表”重要思想为指导，深入贯彻落实科学发展观，坚持稳中求进，加强和改善宏观调控，继续处理好保持经济平稳较快发展、调整经济结构和管理通胀预期的关系，加快推进经济发展方式转变和经济结构调整，着力扩大国内需求特别是消费需求，着力加强自主创新和节能减排，着力深化改革开放，着力保障和改善民生，全面推进社会主义经济建设、政治建设、文化建设、社会建设以及生态文明建设，努力实现经济平稳较快发展和物价总水平基本稳定，保持社会和谐稳定，以经济社会发展的优异成绩迎接党的十八大胜利召开。

　　 今年经济社会发展的主要预期目标是：国内生产总值增长7.5；城镇新增就业900万人以上，城镇登记失业率控制在4.6以内；居民消费价格涨幅控制在4左右；进出口总额增长10左右，国际收支状况继续改善。同时，要在产业结构调整、自主创新、节能减排等方面取得新进展，城乡居民收入实际增长和经济增长保持同步。这里要着重说明，国内生产总值增长目标略微调低，主要是要与“十二五”规划目标逐步衔接，引导各方面把工作着力点放到加快转变经济发展方式、切实提高经济发展质量和效益上来，以利于实现更长时期、更高水平、更好质量发展。提出居民消费价格涨幅控制在4左右，综合考虑了输入性通胀因素、要素成本上升影响以及居民承受能力，也为价格改革预留一定空间。

　　 综合考虑各方面情况，要继续实施积极的财政政策和稳健的货币政策，根据形势变化适时适度预调微调，进一步提高政策的针对性、灵活性和前瞻性。

　　 继续实施积极的财政政策。保持适度的财政赤字和国债规模。今年拟安排财政赤字8000亿元，赤字率下降到1.5左右，其中中央财政赤字5500亿元，代发地方债2500亿元。优化财政支出结构、突出重点，更加注重向民生领域倾斜，加大对教育、文化、医疗卫生、就业、社会保障、保障性安居工程等方面的投入。更加注重加强薄弱环节，加大对“三农”、欠发达地区、科技创新和节能环保、水利、地质找矿等的支持。更加注重勤俭节约，严格控制“三公经费”，大力精简会议和文件，深化公务用车制度改革，进一步降低行政成本。继续控制楼堂馆所建设规模和标准，压缩大型运动会场馆建设投入。全面加强对重点领域、重点部门和重点资金的审计。实施结构性减税。认真落实和完善支持小型微型企业和个体工商户发展的各项税收优惠政策，开展营业税改征增值税试点。继续对行政事业性收费和政府性基金进行清理、整合和规范。加强地方政府性债务管理和风险防范。按照分类管理、区别对待、逐步化解的原则，继续妥善处理存量债务。进一步清理规范地方政府融资平台公司。坚决禁止各级政府以各种形式违规担保、承诺。同时，把短期应对措施和长期制度建设结合起来，严格控制地方政府新增债务，将地方政府债务收支分类纳入预算管理。

　　 继续实施稳健的货币政策。按照总量适度、审慎灵活的要求，兼顾促进经济平稳较快发展、保持物价稳定和防范金融风险。综合运用各种货币政策工具，调节好货币信贷供求，保持社会融资规模合理增长。广义货币预期增长14。优化信贷结构，支持国家重点在建、续建项目和保障性安居工程建设，加强对符合产业政策、有市场需求的企业特别是小型微型企业的信贷支持，切实降低实体经济融资成本。继续严格控制对高耗能、高污染和产能过剩行业的贷款。完善人民币汇率形成机制，增强人民币汇率双向浮动弹性，保持人民币汇率在合理均衡水平上的基本稳定。大力发展外汇市场，丰富外汇产品，为市场主体提供更多的汇率避险工具，管好用好外汇储备。建立健全系统性金融风险防范和监管协调机制，增强抵御风险能力。加强跨境资本流动监控。规范各类借贷行为，引导民间融资健康发展。

　　 全面做好今年的工作，必须坚持突出主题、贯穿主线、统筹兼顾、协调推进，把稳增长、控物价、调结构、惠民生、抓改革、促和谐更好地结合起来。稳增长，就是要坚持扩大内需、稳定外需，大力发展实体经济，努力克服国内外各种不稳定不确定因素的影响，及时解决苗头性、倾向性问题，保持经济平稳运行。控物价，就是要继续采取综合措施，保持物价总水平基本稳定，防止价格走势反弹。调结构，就是要有扶有控，提高经济增长质量和效益，增强发展的协调性和可持续性。惠民生，就是要坚持把保障改善民生作为工作的根本出发点和落脚点，把促进社会公平正义放在更加突出的位置，切实办成一些让人民群众得实惠的好事实事。抓改革，就是要以更大的决心和气力推进改革开放，着力解决影响经济社会长期健康发展的体制性、结构性矛盾，在一些重点领域和关键环节取得新突破。以开放促改革、促发展、促创新。促和谐，就是要正确处理改革、发展、稳定三者关系，积极有效化解各种矛盾和风险隐患，防止局部性问题演变成全局性问题，促进社会和谐稳定。

　　 三、2012年主要任务

　　 （一）促进经济平稳较快发展

　　 扩大内需特别是消费需求是我国经济长期平稳较快发展的根本立足点，是今年工作的重点。

　　 着力扩大消费需求。加快构建扩大消费的长效机制。大力调整收入分配格局，增加中低收入者收入，提高居民消费能力。完善鼓励居民消费政策。大力发展社会化养老、家政、物业、医疗保健等服务业。鼓励文化、旅游、健身等消费，落实好带薪休假制度。积极发展网络购物等新型消费业态。支持引导环保建材、节水洁具、节能汽车等绿色消费。扩大消费信贷。加强城乡流通体系和道路、停车场等基础设施建设。加强产品质量安全监管。改善消费环境，维护消费者合法权益。

　　 不断优化投资结构。保持投资稳定增长，促进投资和消费良性互动。认真落实《国务院关于鼓励和引导民间投资健康发展的若干意见》，出台具有可操作性的实施细则。加强政府投资对结构调整的引领作用，优先保证重点在建、续建项目，有序推进国家重大项目开工建设。把好土地、信贷、节能、环保、安全、质量等准入和审核关，加强对重大项目特别是政府和国有投资项目的监管、督查，提高投资质量和效益。

　　 （二）保持物价总水平基本稳定

　　 这是关系群众利益和经济社会发展全局的重点工作。要在有效实施宏观经济政策、管好货币信贷总量、促进社会总供求基本平衡的基础上，搞好价格调控，防止物价反弹。

　　 增加生产、保障供给。继续把抑制食品价格过快上涨作为稳定物价的重点。落实好“米袋子”省长负责制和“菜篮子”市长负责制，保障主要农产品供给。大中城市要有合理的菜地保有量，稳定和提高本地应季蔬菜自给水平，同周边地区和优势产区协作建设“菜篮子”产品基地。加强重要商品产运销衔接，完善政府储备和商业储备体系，做好主要农产品收储和投放，增强市场调控能力。

　　 搞活流通、降低成本。严格执行蔬菜等鲜活农产品运输绿色通道政策。认真落实对农产品批发市场、集贸市场、社区平价菜店等的扶持政策，鼓励城市连锁超市、高校、大型企业、社区与农产品流通企业、专业合作社、种养大户对接，减少流通环节，增加零售网点，充分发挥流通主渠道作用。深化流通体制改革。扩大物流企业营业税差额纳税试点范围，完善大宗商品仓储设施用地税收政策。调整完善部分农产品批发、零售增值税政策，推动流通标准化、信息化建设。要多管齐下，切实把流通效率提上去、中间成本降下来，真正让生产者和消费者都得到好处。

　　 加强监管、规范秩序。重点加强对食品、药品价格和医疗、通信、教育等服务收费的监督检查，坚决治理交通运输领域乱收费乱罚款，纠正大型零售商业企业违规收费行为，严厉查处发布虚假信息、囤积居奇、操纵价格、恶意炒作等违法行为。把握好舆论导向，正确引导社会预期。

　　 （三）促进农业稳定发展和农民持续增收

　　 在工业化和城镇化发展进程中，要更加重视农业现代化。必须坚持把解决好“三农”问题作为各项工作的重中之重，进一步加大强农惠农富农政策力度，巩固和发展农业农村好形势。

　　 稳定发展农业生产，多渠道增加农民收入。继续开展粮食稳定增产行动，稳定粮食种植面积，着力提高单产。引导农民调整结构，扩大紧缺、优质农产品生产，支持蔬菜、肉蛋奶、水产品等生产。农业补贴要继续增加总量，提高标准，扩大范围，完善机制，新增补贴重点向种养大户、农民专业合作社及各种生产服务组织倾斜。继续提高粮食最低收购价，今年小麦、稻谷最低收购价平均每50公斤分别提高7.4元和16元。健全主产区利益补偿机制，增加粮油、生猪等重要农产品生产大县奖励补助资金。实施新10年农村扶贫开发纲要，按照新的国家扶贫标准，全面做好扶贫开发工作，加大集中连片特殊困难地区扶贫开发力度，让扶贫对象更多地分享改革发展成果。

　　 加快农业科技进步。农业的根本出路在科技。要大力推动农业科技创新，加大对良种繁育、疫病防控、农产品质量安全等关键技术研发和应用的支持力度。加快推进基层农技推广服务体系改革和建设，健全乡镇或区域性农业公共服务机构。完善农业技术补贴制度，促进先进适用农业技术到田到户。建好现代农业示范区，推进高产创建和标准化创建。加快农业机械化步伐。

　　 加强农业农村基础设施建设。今年中央财政用于“三农”的投入拟安排12287亿元，比上年增加1868亿元。要搞好灌区配套改造和小型农田水利建设，大力发展节水农业，加大土地开发整理复垦力度，大规模建设旱涝保收高标准基本农田。加快中小河流治理、小型水库除险加固和山洪地质灾害综合防治。加强农村水电路气以及文化体育等基础设施建设，推进农村环境治理，加快农村危房改造，继续改善农村生产生活条件。

　　 深化农村改革。坚持农村基本经营制度不动摇。要认真搞好土地确权登记颁证。土地承包经营权、宅基地使用权、集体收益分配权是法律赋予农民的财产权利，任何人都不能侵犯。加强土地承包经营权流转管理和服务，发展适度规模经营。严格保护耕地。制定出台农村集体土地征收补偿条例。扶持发展农民专业合作社、产业化龙头企业，开展多种形式的农业社会化服务，发展农业保险，提高农业产业化、组织化程度。深化农村综合改革。推进国有农场、林场体制改革，继续深化集体林权制度改革。

　　 （四）加快转变经济发展方式

　　 解决发展不平衡、不协调、不可持续的问题，关键在于加快转变经济发展方式，推进经济结构战略性调整，这既是一个长期过程，也是当前最紧迫的任务。

　　 促进产业结构优化升级。推动战略性新兴产业健康发展。建立促进新能源利用的机制，加强统筹规划、项目配套、政策引导，扩大国内需求，防止太阳能、风电设备制造能力的盲目扩张。发展新一代信息技术，加强网络基础设施建设，推动三网融合取得实质性进展。大力发展高端装备制造、节能环保、生物医药、新能源汽车、新材料等产业。扩大技改专项资金规模，促进传统产业改造升级。以汽车、钢铁、造船、水泥等行业为重点，控制增量，优化存量，推动企业兼并重组，提高产业集中度和规模效益。落实并完善促进小型微型企业发展的政策，进一步减轻企业负担，激发科技型小型微型企业发展活力。实施有利于服务业发展的财税、金融政策，支持社会资本进入服务业，促进服务业发展提速、比重提高、水平提升。

　　 推进节能减排和生态环境保护。节能减排的关键是节约能源，提高能效，减少污染。要抓紧制定出台合理控制能源消费总量工作方案，加快理顺能源价格体系。综合运用经济、法律和必要的行政手段，突出抓好工业、交通、建筑、公共机构、居民生活等重点领域和千家重点耗能企业节能减排，进一步淘汰落后产能。加强用能管理，发展智能电网和分布式能源，实施节能发电调度、合同能源管理、政府节能采购等行之有效的管理方式。优化能源结构，推动传统能源清洁高效利用，安全高效发展核电，积极发展水电，加快页岩气勘查、开发攻关，提高新能源和可再生能源比重。加强能源通道建设。深入贯彻节约资源和保护环境基本国策。开展节能认证和能效标识监督检查，鼓励节能、节水、节地、节材和资源综合利用，大力发展循环经济。加强环境保护，着力解决重金属、饮用水源、大气、土壤、海洋污染等关系民生的突出环境问题。努力减少农业面源污染。严格监管危险化学品。今年在京津冀、长三角、珠三角等重点区域以及直辖市和省会城市开展细颗粒物（PM2.5）等项目监测，2015年覆盖所有地级以上城市。推进生态建设，建立健全生态补偿机制，促进生态保护和修复，巩固天然林保护、退耕还林还草、退牧还草成果，加强草原生态建设，大力开展植树造林，推进荒漠化、石漠化、坡耕地治理，严格保护江河源、湿地、湖泊等重要生态功能区。加强适应气候变化特别是应对极端气候事件能力建设，提高防灾减灾能力。坚持共同但有区别的责任原则和公平原则，建设性推动应对气候变化国际谈判进程。我们要用行动昭告世界，中国绝不靠牺牲生态环境和人民健康来换取经济增长，我们一定能走出一条生产发展、生活富裕、生态良好的文明发展道路。

　　 促进区域经济协调发展。实施区域发展总体战略和主体功能区规划，充分发挥各地特色和优势，进一步提高区域发展的协调性和基本公共服务均等化水平。认真落实西部大开发新10年的政策措施，加大实施中部地区崛起战略的力度，加快推进东北地区等老工业基地振兴，积极支持东部地区转型发展、在更高层次上参与国际竞争与合作。要加大对革命老区、民族地区、边疆地区和贫困地区的扶持力度。更好地发挥经济特区、上海浦东新区、天津滨海新区在改革开放中先行先试的重要作用。制定和实施海洋发展战略，促进海洋经济发展。加强和完善跨区域合作机制，消除市场壁垒，促进要素流动，引导产业有序转移，推动区域经济良性互动、协调发展。

　　 积极稳妥推进城镇化。要遵循城市发展规律，从各地实际出发，促进大中小城市和小城镇协调发展。根据资源环境和人口承载能力，优化全国生产力布局，形成合理的城镇体系和与国土规模、资源分布、发展潜力相适应的人口布局。各类城市都要夯实经济基础，创造就业机会，完善基础设施，改善人居环境，加强管理服务，提升城镇化质量和水平。更加注重把在城镇稳定就业和居住的农民工有序转变为城镇居民；放宽中小城市落户条件，合理引导人口流向，让更多农村富余劳动力就近转移就业。加强对农民工的人文关怀和服务，着力解决农民工在就业服务、社会保障、子女入园上学、住房租购等方面的实际问题，逐步将城镇基本公共服务覆盖到农民工。关爱留守儿童、留守妇女和留守老人。让农民无论进城还是留乡，都能安居乐业、幸福生活。

　　 （五）深入实施科教兴国战略和人才强国战略

　　 大力发展科技、教育事业，培养高素质的人才队伍，是国家强盛、民族复兴的必由之路。

　　 坚持优先发展教育。中央财政已按全国财政性教育经费支出占国内生产总值的4编制预算，地方财政要相应安排，确保实现这一目标。教育经费要突出保障重点，加强薄弱环节，提高使用效益。深入推进教育体制改革，加强教师队伍建设，大力实施素质教育，逐步解决考试招生、教育教学等方面的突出问题。推进学校民主管理，逐步形成制度。促进义务教育均衡发展，资源配置要向中西部、农村、边远、民族地区和城市薄弱学校倾斜。继续花大气力推动解决择校、入园等人民群众关心的热点难点问题。农村中小学布局要因地制宜，处理好提高教育质量和方便孩子们就近上学的关系。办好农村寄宿学校，实施好农村义务教育学生营养改善计划。加强校车和校园安全管理，确保孩子们的人身安全。加强学前教育、继续教育和特殊教育，建设现代职业教育体系。办好民族教育。高等教育要与经济社会发展和国家战略需要紧密结合，提高教育质量和创新能力。完善国家助学制度，逐步将中等职业教育免学费政策覆盖到所有农村学生，扩大普通高中家庭经济困难学生资助范围。大力发展民办教育，鼓励和引导社会资本进入各级各类教育领域。教育寄托着人民的希望，关系国家的未来，我们一定要把这项事业办得更好！

　　 大力推进科技创新。加强国家创新体系建设。深化科技体制改革，推动企业成为技术创新主体，促进科技与经济紧密结合。支持企业加强研发中心建设，承担国家和地区重大科技项目。引导科研机构、高等院校的科研力量为企业技术创新服务，更好地实现产学研有机结合，提高科技成果转化和产业化水平。推动基础研究和前沿技术研究，提高原始创新能力。完善科技评价和奖励制度。倡导学术诚信，鼓励独立思考，保障学术自由，弘扬科学精神。坚定不移地实施国家知识产权战略。

　　 全面加强人才工作。深化人才体制改革，大力培养造就高水平创新创业人才、青年人才和急需紧缺人才，引进高层次人才。完善人才培养、任用、评价、激励机制。努力营造人才辈出、人尽其才、才尽其用的良好社会环境。

　　 （六）切实保障和改善民生

　　 实现好、维护好、发展好最广大人民的根本利益是以人为本理念的具体体现。要把保障和改善民生作为政府工作的重要任务。

　　 千方百计扩大就业。就业是关系国家发展和人民福祉的大事。今年就业压力仍然很大，各级政府务必坚持就业优先战略，继续实施更加积极的就业政策。重点扶持就业容量大的服务业、创新型科技企业和小型微型企业，创造更多就业岗位。鼓励以创业带动就业。抓好高校毕业生、农民工和城镇就业困难人员就业，加强退役军人技能培训与就业安置工作。鼓励高校毕业生投身农村、基层、中西部地区建设。加强职业培训和公共就业服务工作。加快建立健全统一规范灵活的人力资源市场。积极构建和谐劳动关系，加强对劳务派遣的规范管理，开展劳动关系争议排查，加强劳动监察和调解仲裁，维护劳动者合法权益。

　　 加快完善社会保障体系。今年年底前实现新型农村社会养老保险和城镇居民社会养老保险制度全覆盖。扩大各项社会保险覆盖面。增加企业退休人员基本养老金。加强城乡低保和社会救助工作，加快发展社会福利事业和慈善事业。加强各项社会保障制度衔接。多渠道增加社会保障基金，加强社会保险基金、社会保障基金投资监管，实现保值增值。加强社保服务能力建设，有条件的地方可对各类社保经办机构进行整合归并，有些服务可委托银行、商业保险机构代办。加快全国统一的社会保障卡发放。

　　 大力推进医药卫生事业改革发展。加快健全全民医保体系，巩固扩大基本医保覆盖面，提高基本医疗保障水平和管理服务水平。城镇居民医保和新农合补助标准提高到每人每年240元。全面推开尿毒症等8类大病保障，将肺癌等12类大病纳入保障和救助试点范围。巩固完善基本药物制度，加强基层医疗卫生服务体系建设。推进公立医院改革，实行医药分开、管办分开，破除以药补医机制。鼓励引导社会资本办医，加快形成对外开放的多元办医格局。充分调动医务工作者积极性，建立和谐的医患关系。加强公共卫生服务，预防控制严重威胁群众健康的重大传染病、慢性病、职业病。加强药品安全工作。扶持和促进中医药和民族医药事业发展。

　　 全面做好人口和计划生育工作。继续稳定低生育水平，综合治理出生人口性别比偏高问题，提高出生人口质量。加快实现计划生育优质服务全覆盖，将免费孕前优生健康检查试点范围扩大到60的县（市、区）。提高农村部分计划生育家庭奖励扶助、特殊扶助标准。加强流动人口计划生育服务管理。做好妇女儿童工作，扩大农村妇女宫颈癌、乳腺癌免费检查覆盖面，提高妇女儿童发展和权益保障水平。进一步完善残疾人社会保障体系和服务体系。积极发展老龄事业，加快建设社会养老服务体系，努力让城乡老年人都老有所养，幸福安度晚年。

　　 继续搞好房地产市场调控和保障性安居工程建设。严格执行并逐步完善抑制投机、投资性需求的政策措施，进一步巩固调控成果，促进房价合理回归。继续推进保障性安居工程建设，在确保质量的前提下，基本建成500万套，新开工700万套以上。抓紧完善保障性住房建设、分配、管理、退出等制度。采取有效措施，增加普通商品住房供给。加快建设城镇住房信息系统，改革房地产税收制度，促进房地产市场长期平稳健康发展。

　　 加强和创新社会管理。加强社会矛盾化解、社会管理创新、公正廉洁执法。强化政府社会管理和公共服务职能。提高城乡基层群众性自治组织的自治能力。发挥社会组织在社会管理中的积极作用。积极稳妥推进户籍管理制度改革，推动实行居住证制度，为流动人口提供更好服务。以信息共享、互联互通为重点，加快建设国家电子政务网。大力推进政务诚信、商务诚信、社会诚信建设，构建覆盖全社会的征信系统。加强和改进互联网管理，营造健康的网络环境。健全重大决策社会稳定风险评估机制和突发事件应急管理机制。实施安全发展战略，加强安全生产监管，防止重特大事故发生。深入开展打击侵犯知识产权和制售假冒伪劣商品行动。增强食品安全监管能力，提高食品安全水平。加强和改进信访工作，健全群众利益诉求表达机制。加强法律服务和法律援助。严密防范和依法打击违法犯罪活动，保障人民群众生命财产安全。

　　 （七）促进文化大发展大繁荣

　　 文化是人类的精神家园，优秀文化传承是一个民族生生不息的血脉。要提供优质丰富的文化产品，不断满足人民群众的精神文化需求。深入推进社会主义核心价值体系建设。加强社会公德、职业道德、家庭美德和个人品德教育，做好青少年思想道德教育工作，努力形成知荣辱、讲正气、守诚信、作奉献、促和谐的良好风尚。大力发展公益性文化事业。以农村和中西部地区为重点，加强基层文化设施建设。推动哲学社会科学繁荣发展，积极发展新闻出版、广播影视、文学艺术和档案事业。加强文化遗产保护，繁荣发展少数民族文化事业。深化文化体制改革，继续推动经营性文化单位转企改制。提高文化产业规模化、集约化、专业化水平，推动文化产业成为国民经济支柱性产业。深入开展对外人文交流，促进中外文化相互借鉴。广泛开展全民健身活动，增强人民体质，促进体育事业和体育产业协调发展。中华文化具有强大的向心力和震撼力，当代中华儿女一定要肩负起弘扬中华文化的历史重任。

　　 （八）深入推进重点领域改革

　　 改革开放是决定中国前途命运的正确抉择。必须按照科学发展观要求，尊重群众首创精神，大胆探索，以更大决心和勇气继续全面推进经济体制、政治体制等各项改革，破解发展难题。当前和今后一段时期，改革的重点领域和关键环节是：进一步转变政府职能，完善宏观调控体系，理顺政府与市场的关系，更好地发挥市场配置资源的基础性作用；推进财税体制改革，理顺中央与地方及地方各级政府间财政分配关系，更好地调动中央和地方两个积极性；深化土地、户籍、公共服务改革，理顺城市与农村的关系，推动工业化、城镇化和农业现代化协调发展；推进社会事业、收入分配等改革，理顺经济与社会发展的关系，有效保障社会公平正义；推进依法行政和社会管理创新，理顺政府与公民和社会组织的关系，建设服务、责任、法治、廉洁政府。今年改革的重点任务是：

　　 深化财税金融体制改革。完善分税制，健全转移支付制度，提高一般性转移支付规模和比例。完善县级基本财力保障机制。稳步推进地方财政预算、决算公开。深化国库集中收付、政府采购及国债管理制度改革。健全消费税制度。全面深化资源税改革，扩大从价计征范围。深入推进国有控股大型金融机构改革，规范发展小型金融机构，健全服务小型微型企业和“三农”的体制机制。推动实施银行业新监管标准。推进建立存款保险制度。深化政策性金融机构改革。健全完善新股发行制度和退市制度，强化投资者回报和权益保护。积极发展债券市场。推进保险业改革发展。深化利率市场化改革。稳步推进人民币资本项目可兑换，扩大人民币在跨境贸易和投资中的使用。

　　 推动多种所有制经济共同发展。毫不动摇地巩固和发展公有制经济，毫不动摇地鼓励、支持、引导非公有制经济发展。深入推进国有经济战略性调整，完善国有资本有进有退、合理流动机制。研究推进铁路、电力等行业改革。完善和落实促进非公有制经济发展的各项政策措施，打破垄断，放宽准入，鼓励民间资本进入铁路、市政、金融、能源、电信、教育、医疗等领域，营造各类所有制经济公平竞争、共同发展的环境。

　　 深化价格改革。稳妥推进电价改革，实施居民阶梯电价改革方案，完善水电、核电及可再生能源定价机制。逐步理顺煤电价格关系。完善成品油价格改革，推进天然气价格改革。实行最严格的水资源管理制度，合理制定和调整各地水资源费征收标准，推进农业水价综合改革。开展碳排放和排污权交易试点。

　　 深化收入分配制度改革。抓紧制定收入分配体制改革总体方案。努力提高居民收入在国民收入分配中的比重，提高劳动报酬在初次分配中的比重。完善工资制度，建立工资正常增长机制，稳步提高最低工资标准。创造条件增加居民财产性收入。建立公共资源出让收益的全民共享机制。加大对高收入者的税收调节力度，严格规范国有企业、金融机构高管人员薪酬管理，扩大中等收入者比重，提高低收入者的收入，促进机会公平。规范收入分配秩序，有效保护合法收入，坚决取缔非法收入，尽快扭转收入差距扩大的趋势。

　　 积极稳妥推进事业单位分类改革。科学划分事业单位类别，分类指导、分业推进、分级组织、分步实施，深化事业单位管理体制和人事、收入分配、社会保障制度改革。

　　 加快推进政府改革。扩大社会主义民主，依法实行民主选举、民主决策、民主管理、民主监督，保障人民的知情权、参与权、表达权和监督权。全面贯彻依法治国基本方略，尊重和维护宪法和法律的权威，严格依法行政，坚决纠正有法不依、执法不严、违法不究、粗暴执法、渎职失职和执法腐败等行为。加强廉政建设，深入开展反腐败斗争，扎实推进惩治和预防腐败体系建设的各项长期性、基础性工作，着力解决人民群众反映强烈的突出问题。严格依法设定、实施、清理、规范行政审批事项。严禁领导干部插手政府采购、工程招标、土地矿业权拍卖等经济活动。严格执行领导干部廉洁从政各项规定。加强行政监督、民主监督、舆论监督。坚决查处各类违纪违法案件，严厉惩治腐败分子。

　　 （九）努力提高对外开放的质量和水平

　　 我国对外开放已进入新的阶段，进出口贸易、双向投资的地位和作用发生了深刻变化。必须适应新的形势，创新对外经济工作思路，转变对外经济发展方式，提升开放型经济水平，形成开放型经济新格局。

　　 保持对外贸易稳定发展。我们强调扩大内需，但决不能忽视外需对我国经济发展的重要作用。要保持外贸政策基本稳定。稳定出口退税政策，扩大贸易融资和信用保险，改进海关、质检、外汇等方面的监管和服务，帮助企业克服订单不足、成本升高、摩擦增多等多重困难和压力。要加快转变外贸发展方式。深入实施科技兴贸、以质取胜和市场多元化战略，支持企业培育自主品牌、营销网络和研发中心，引导加工贸易向产业链高端延伸、向中西部转移。巩固美日欧传统市场，开拓新兴市场。稳定劳动密集型产品出口，扩大高技术、高附加值产品出口，控制高耗能、高污染产品出口。大力发展服务贸易，承接服务外包。制定加强进口、促进贸易平衡的指导意见，完善进口政策，搭建更多的进口促进平台，推动进出口平衡发展。

　　 提高利用外资质量。坚持积极有效利用外资的方针，更加注重优化结构和提高质量。实施新修订的外商投资产业指导目录，引导外资更多投向先进制造业、高新技术产业、节能环保产业、现代服务业和中西部地区。

　　 实施“走出去”战略。我国正处于对外投资加快发展的重要阶段，要加强宏观指导，强化政策支持，简化审批手续，健全服务保障。引导各类所有制企业有序开展境外能源、原材料、农业、制造业、服务业、基础设施等领域投资合作和跨国并购。创新境外经贸合作区发展模式，支持“走出去”的企业相互协同、集群发展。规范发展对外劳务合作。放宽居民境外投资限制。加强对外投资风险管理，维护我境外企业人员和资产安全。

　　 参与全球经济治理和区域合作。努力保持与发达国家经贸关系稳定发展，全面深化与发展中国家的互利合作。继续推进自贸区建设和区域经济一体化进程。积极参与二十国集团等全球经济治理机制建设，加强与主要经济体宏观经济政策协调，反对各种形式的保护主义，继续在多哈回合谈判、国际金融体系改革中发挥建设性作用。

　　 各位代表！

　　 我国是统一的多民族国家，各民族共同团结奋斗、共同繁荣发展，国家才能兴旺发达。要坚持和完善民族区域自治制度，认真贯彻落实中央支持少数民族和民族地区发展的政策措施，大力实施扶持人口较少民族发展、推进兴边富民行动和发展少数民族事业规划。坚定不移地巩固和发展平等团结互助和谐的社会主义民族关系。

　　 认真贯彻党的宗教工作基本方针。维护宗教团体、宗教界人士和信教群众的合法权益，充分发挥他们在促进经济发展、文化繁荣、社会和谐中的积极作用。

　　 全面贯彻党的侨务政策。维护海外侨胞和归侨侨眷合法权益，支持他们积极参与祖国现代化建设与和平统一大业。

　　 各位代表！

　　 巩固的国防和强大的军队，是维护国家主权、安全和发展利益的坚强后盾。要着眼全面履行新世纪新阶段军队历史使命，全面加强军队革命化现代化正规化建设，不断提高以打赢信息化条件下局部战争能力为核心的完成多样化军事任务的能力。加强思想政治建设，坚持党对军队绝对领导的根本原则和人民军队的根本宗旨。积极开展信息化条件下军事训练。加快全面建设现代后勤步伐。大力提高国防科技和武器装备自主创新能力。着力培养高素质新型军事人才。积极稳妥地实施国防和军队改革。坚持依法治军、从严治军。全面建设现代化武装警察力量。加强国防动员和后备力量建设。坚决完成反恐维稳、处置突发事件、抢险救灾等任务。坚持军民结合、寓军于民，走中国特色军民融合式发展路子。巩固发展军政军民团结。

　　 各位代表！

　　 香港、澳门与祖国休戚相关、荣辱与共。我们将坚定不移地贯彻“一国两制”、“港人治港”、“澳人治澳”、高度自治的方针，全力支持香港、澳门发展经济，改善民生，推进民主。支持特区政府积极应对国际经济风险挑战，维护经济金融稳定和长期繁荣发展。衔接和落实好支持港澳经济社会发展的系列政策措施，大幅提升内地对港澳服务贸易开放水平，加快推进港珠澳大桥等基础设施建设和对接，深化合作，支持港澳参与国际和区域经济合作。支持香港巩固和提升国际金融、贸易、航运中心地位，建设离岸人民币业务中心。支持澳门建设世界旅游休闲中心，推进横琴新区建设，促进经济适度多元发展。我们相信，有伟大祖国作为坚强后盾，香港、澳门同胞一定能够把自己的家园建设得更加美好！

　　 过去的一年，两岸关系经受了严峻考验，取得了积极进展。反对“台独”、认同“九二共识”，巩固交流合作成果，促进两岸关系和平发展，日益成为两岸同胞的共同意愿。新的一年，我们要继续坚持中央对台工作的大政方针，增强两岸关系发展的政治、经济、文化和民意基础，拓展两岸关系和平发展新局面。要全面深化经济金融合作，推动两岸经济合作框架协议后续商谈取得新进展。加快海峡西岸经济区建设。积极扩大各界往来，开展文化、教育等交流，使两岸同胞联系更紧密，感情更贴近，利益更融合。全体中华儿女要更加紧密地团结起来，为完成祖国统一大业、实现中华民族伟大复兴而努力奋斗！

　　 各位代表！

　　 新的一年，外交工作要更好地服务于改革开放和社会主义现代化建设大局，为促进世界经济增长、维护和平稳定作出更大贡献。我们将继续深化同周边国家的睦邻友好关系，积极参与周边各种合作机制，推动区域合作深入发展，共同营造和平稳定、平等互信、合作共赢的地区环境。我们将与广大发展中国家加强团结合作，深化传统友谊，扩大互利合作，推动实现联合国千年发展目标，维护发展中国家的正当权益和共同利益。我们将加强与各大国的战略对话，增进战略互信，拓展合作领域，推进相互关系长期稳定健康发展。我们将积极参与多边事务和全球治理，推动国际秩序朝着更加公正合理的方向发展。我们将坚定不移地走和平发展道路，坚持独立自主的和平外交政策，奉行互利共赢的开放战略，同世界各国一道，为促进人类文明进步，增进各国人民的福祉和建设一个持久和平、共同繁荣的和谐世界而不懈努力！

　　 各位代表！

　　 回顾过去，我们拼搏奋进，取得显著成就；展望未来，我们任重道远，仍须不懈努力。让我们紧密团结在以胡锦涛同志为总书记的党中央周围，解放思想，开拓创新，扎实工作，奋力开创社会主义现代化建设新局面！
